# Assessing Knowledge and Awareness of Attention Deficit Hyperactivity Disorder Among Sudanese Medical Students

**DOI:** 10.1192/bjo.2025.10143

**Published:** 2025-06-20

**Authors:** Esra Tayfour, Suad Abdelwahab, Hanan Mustafa, Tarteel Abdelmagid, Danya Ibrahim

**Affiliations:** ^1^Faculty of Medicine, Ahfad University for Women, Khartoum, Sudan; ^2^Faculty of Medicine, University of Khartoum, Khartoum, Sudan

## Abstract

**Aims:** This study aimed to assess the knowledge and awareness of ADHD among medical students at a Sudanese university.

**Methods:** This cross-sectional study was conducted at Ahfad University for Women (AUW) from December 2019 to February 2020. A self-administered questionnaire was distributed to 212 female medical students in their fifth and sixth years to assess their knowledge and awareness of ADHD.

**Results:** Out of the 212 students approached, 131 (61.8%) completed the questionnaire. Participants’ ages ranged from 23–25 years. Most students (64.2%, n=82) were familiar with ADHD through their medical curriculum. While 71.3% (n=93) correctly identified key symptoms of ADHD, only 32.6% (n=43) were aware of evidence-based management strategies. Additionally, 62.3% (n=82) expressed dissatisfaction with the amount of information provided in their psychiatry courses.

**Conclusion:** This study highlights a significant knowledge gap among medical students regarding ADHD, particularly in evidence-based management. The findings underscore the need for improvements in the psychiatry curriculum at Ahfad University for Women to better equip future doctors to address ADHD in clinical practice.

